# An Experiential Learning Based Design Program in Rehabilitation Engineering

**DOI:** 10.1007/s43683-022-00091-2

**Published:** 2022-11-14

**Authors:** Sudeshna Pal, Mark Steiner, Alain J. Kassab, Patrick Pabian, Adam Golden, Edward A. Ross

**Affiliations:** 1 Department of Mechanical and Aerospace Engineering, College of Engineering and Computer Sciences, University of Central Florida, Orlando, FL, USA;; 2 Department of Physical Therapy, College of Health Sciences, University of Kentucky, Lexington, KY, USA;; 3 Department of Internal Medicine, College of Medicine, University of Central Florida, Orlando, FL, USA

## CHALLENGE STATEMENT

In recent years, Biomedical Engineering (BME) undergraduate programs have adopted clinical immersion (CI) programs in their curriculum to provide students with better exposure to clinical settings and engineering needs in medicine. Most of these CI programs are geared toward the broad field of BME.^7^ This article describes an experiential learning based design program focused on the BME subfield of Rehabilitation Engineering (RE). RE is a sub-discipline of BME that applies engineering principles in the design and development of devices that assist, improve, or restore lost functionalities of the disabled.^8^ The program elaborated in this article includes didactic and experiential coursework structured to inspire and educate engineering students on a BME career path in RE.

Experiential learning, as described by Kolb, is a learning process where knowledge is created through the four stages of experience, observation, reflection and action.^5^ In BME education, some of the activities in CI courses align with the experiential learning model. These CI courses are beneficial in training students to be better prepared and effective in solving real-world problems, improving the application of classroom knowledge to real-life settings, and training students to identify suitable user-based design needs in clinical settings.^1,7,11^ Recently, the emphasis placed by the US Food and Drug Administration (FDA) on user needs identification through direct interactions with clinicians, users and clinical staff for efficient early-stage medical device development has driven many BME undergraduate programs to incorporate CI courses prior to teaching BME capstone design.^2–10,12^

**Figure 1:**
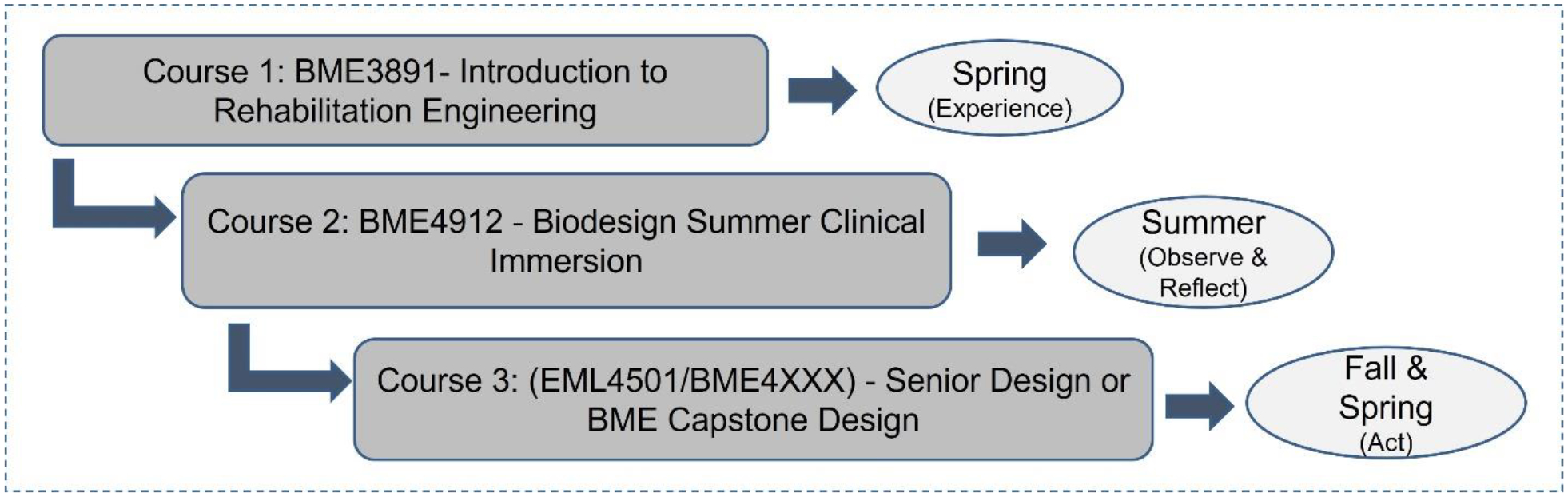
Experiential structure and course sequence of the BPRE program

The need for BME undergraduate programs that focus on the RE sub-discipline of BME is also significant. The National Institute of Disability, Independent Living and Rehabilitation Research estimates that the people with disabilities population is 13.4% of the total US population.^2^ A focused undergraduate program in RE that taps into the advantages of an experiential learning model can benefit this field through skilled workforce generation and narrow the gap between design innovations and the needs of the RE healthcare industry. This *teaching tips* article describes such an interdisciplinary, experiential learning based design program entitled “A Biodesign program in rehabilitation engineering (BPRE)” that was developed and deployed to train engineers in the RE field at the ABET (Accreditation Board for Engineering and Technology) accredited institution of the authors as part of an NIH R25 grant. The program has been successfully offered for three years from spring 2019 to 2022 (current) with a one-year gap due to the pandemic. The BPRE program differs from most CI courses in undergraduate BME programs in its focus on RE, and also in its experiential structure that consists of multiple courses.^5,7^ The program structure follows the four stages of experiential learning, and first inspires and trains students in the RE field through an introductory course (“experience” stage) team-taught by engineering and clinical faculty from physical therapy (PT) and the college of medicine, a component absent in many undergraduate CI programs.^5^ The students are then exposed to summer clinical rotations in RE (“observe and reflect” stage), which is followed by the realization of identified engineering needs in RE through a design course (“act” stage) (Fig. 1).

## NOVEL INITIATIVE

As part of the BPRE program, two new BME undergraduate courses in RE were developed in Spring 2019 primarily by engineering faculty with collaboration from PT and medicine faculty. The PT and medicine faculty supported these courses by providing lectures on clinical perspectives of rehabilitation, facilitating student visits in different clinical assessment laboratories on campus, facilitating many of the CI sites and providing clinical expertise and insights to students during all three stages of the program.

### Course 1:

**Figure 2:**
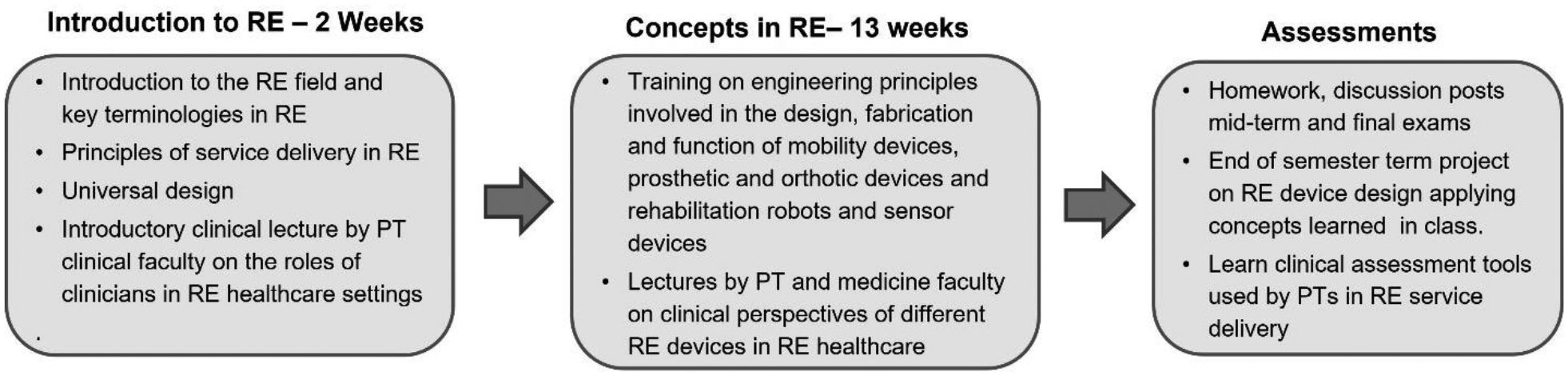
Breakdown of the topics covered and activities in the introductory course in RE

The first BME course developed for the program was an introductory three-credit technical elective course entitled “Introduction to Rehabilitation Engineering” (Fig. 2) for junior and senior undergraduate engineering students. The course was offered in the Spring of each year. The course had a modular structure (Supplemental Table I). In the first two weeks of the course, an introduction to key terminologies in RE, principles of RE service delivery and universal design principles were discussed. This was followed by focused lectures on engineering principles involved in the design, fabrication and function of the different classes of rehabilitative and assistive devices. One to two clinical lectures by PT and medicine faculty were integrated alongside the regular engineering lectures for each class of rehabilitative devices covered to help students better understand the clinician’s perspective on the prescription and use of these devices in RE healthcare settings. During the final four weeks of the course, students worked in teams of 2 on a device design term project applying concepts learned in the class. The final weeks also included visits to PT labs to understand the different clinical assessment tools used in RE. University Institutional Review Board (IRB) approved pre and post surveys were administered to gage the impact of the course on student interest in the discipline. All course modules, assignments and surveys were available to students through the learning management system Canvas^®^ (Salt Lake City, Utah).

### Course 2:

**Figure 3:**
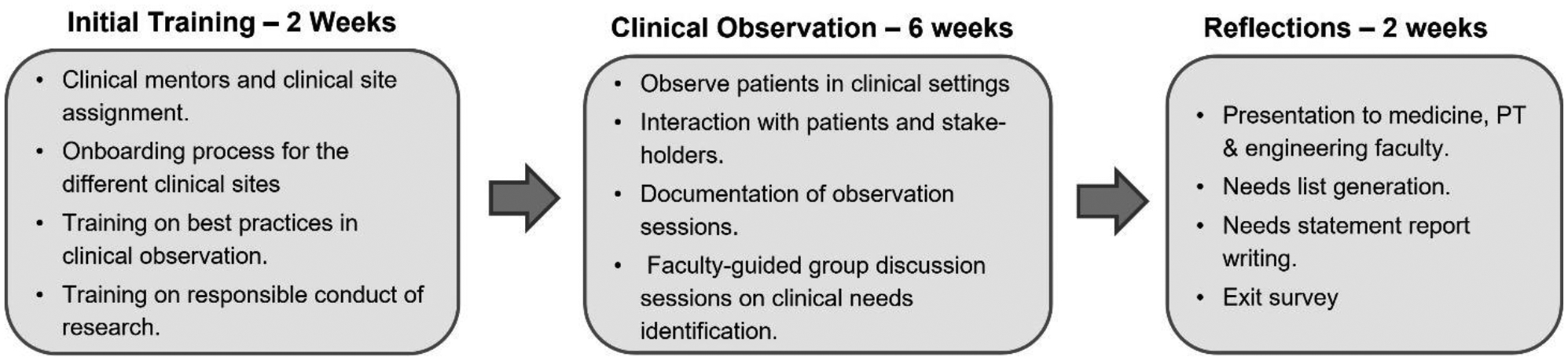
Breakdown of the activities in the clinical immersion course

A sequential one-credit internship-based CI BME course, “Biodesign Summer Clinical Immersion”, in RE was developed for summer of each year. Rising seniors in undergraduate engineering programs who successfully completed the course 1 were selected into this course. Student selection was based on GPA, student performance in course 1 and student motivation and interest gaged through student submitted statements of purpose. This 10-week course (Fig. 3) involved two weeks of initial training and onboarding at the clinics, followed by six-weeks of full-time clinical observations at different rehabilitation centers, prosthetics and PT clinics. The final two weeks focused on reflections and technical report writing. In the six-week CI period, students were placed in teams of two and paired with clinical mentors while they engaged in observation sessions at the different sites. The students met for a weekly two to three hour long instructor-led session, where instruction on best practices in shadowing; efficient identification of clinical needs of patients, caretakers or healthcare providers(needs finding); ways to objectively compare and select clinical needs that may have engineering solutions (needs screening); and final reflections report writing (needs statement) were provided.^14^ At the end of each bi-weekly rotation, students engaged in presentation and discussion sessions with the engineering, PT and medicine faculty for further refinement of the identified clinical needs. At the end of CI, students submitted a detailed needs statement report synthesizing disease and disability information with the solutions requirements for the top five identified needs from each rotation site they visited. An IRB approved exit survey was conducted to gage the learning outcomes from the CI experience of the students at the end of this course.

### BME Design Course:

Based on clinical needs identified during the CI summer course, and after discussions with engineering design, medicine, and PT faculty, three to four needs were pursued as BME capstone design projects the following Fall and Spring semesters after the CI each year. These design projects were offered under the existing structure of the engineering design (senior design) course in the ABET-accredited mechanical engineering program at the University of Central Florida and were funded by the BPRE program through the NIH R25 grant. The design projects were open to all engineering students from the introductory RE course (course 1) and the summer internship course (course 2) and to other interested senior students in the engineering program.

## REFLECTION

**Figure 4:**
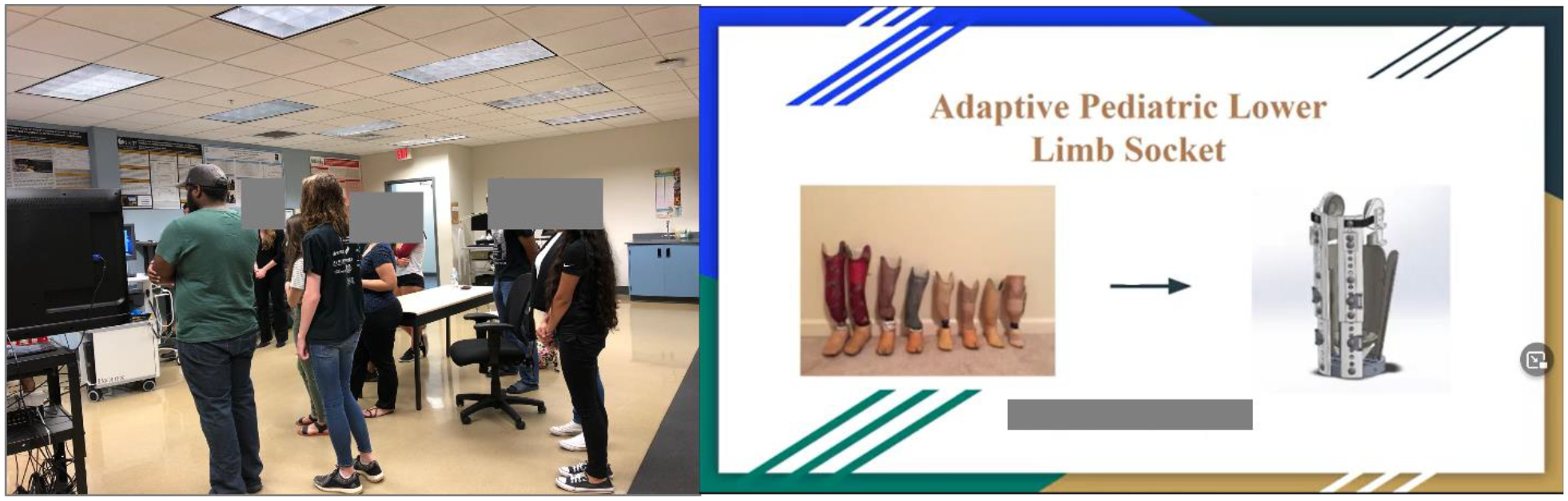
Examples of a PT clinical assessment laboratory session and a student term project from the introductory RE course (course1)

The BPRE program courses were designed to provide undergraduate engineering students (with BME interests) a continued and experiential exposure in RE to inspire them toward careers and innovations in the RE field.^14^ It is evident from pedagogical literature that such continued, interdisciplinary exposures promote gradual buildup of interdisciplinary skills necessary to thrive in BME fields.^10^ Figure 4 highlights the experiential aspect of the first introductory course (course 1), a team-taught PT clinical assessment laboratory session (left) and, a student term project presentation on device design based on concepts learned in class (right).^5^

**Figure 5:**
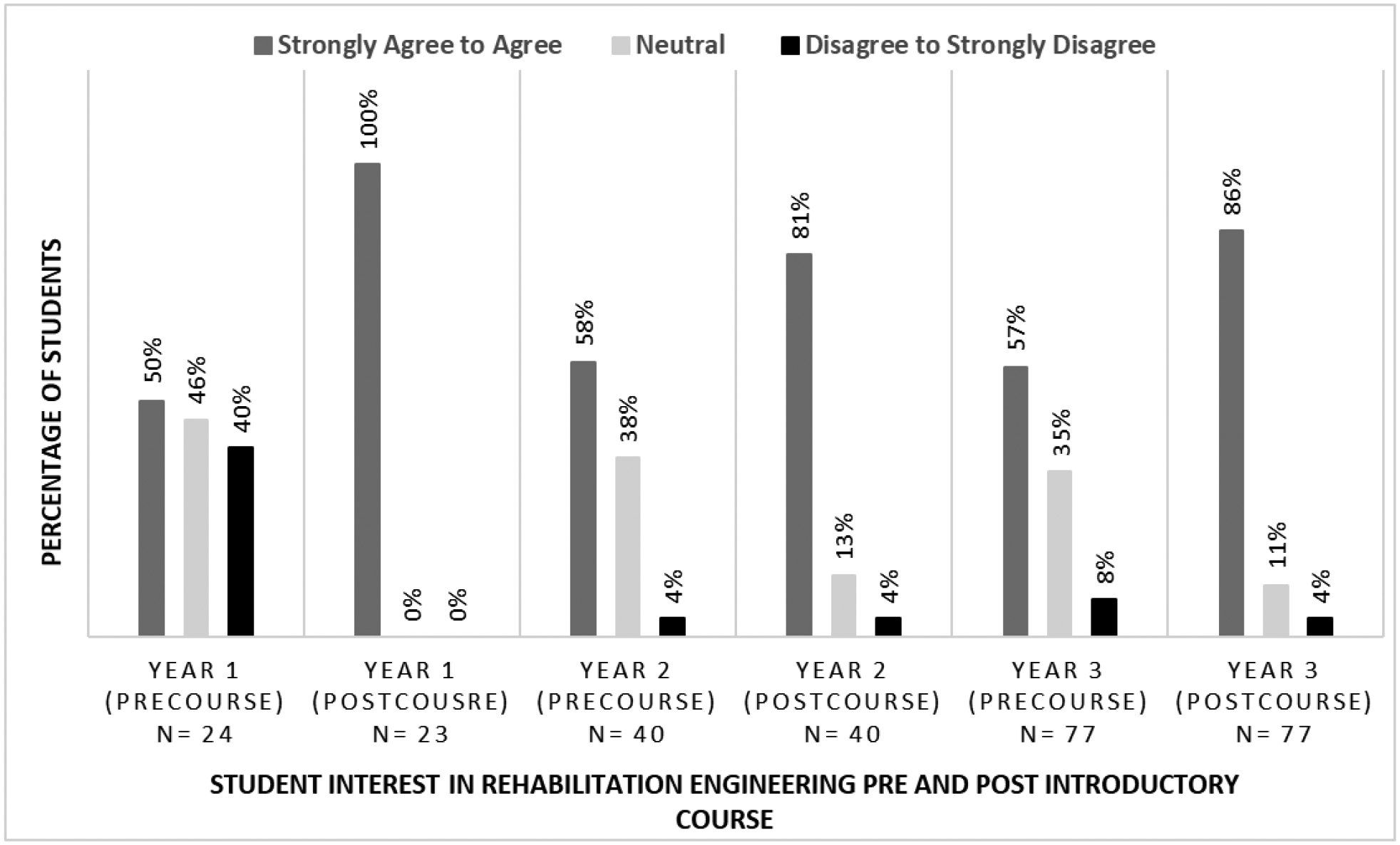
Pre to post-course survey results for student interest in rehabilitation engineering in the introductory RE course (course1)

Student interest in RE after completion of course 1 was assessed through a pre and post course survey question, the results of which are summarized in Fig. 5 (96% students participated in the surveys over three years). As evident in Fig. 5, student interest in RE increased by 23 to 50% from pre to post course over the three-year period. Student enrollment has also tripled by year 3, a positive indicator of the course impact on students with BME interests.

Additionally, students’ perception on the course content and impact were also assessed through post-course survey questions in course 1 (Supplemental Table II). Data showed that majority of the students (87 – 98%) found the course structure suitable for individuals seeking RE careers. More than half of the class (50 – 69%) was interested in a RE BME capstone senior design project, or in a standalone BME capstone course after course completion, while 77 to 95% of the students were willing to consider RE as a career option after completing course 1. Both pre and post course survey data and student comments (below) demonstrate the positive impact of course 1 in building student interest in RE.

“I really enjoyed the guest lectures and the research lab visit! This course truly did develop my interest in the field and has gotten me to consider a future in rehabilitation engineering”.

“Very interesting course, I am glad I was able to take it. It has opened new jobs that I did not know were available”.

**Figure 6:**
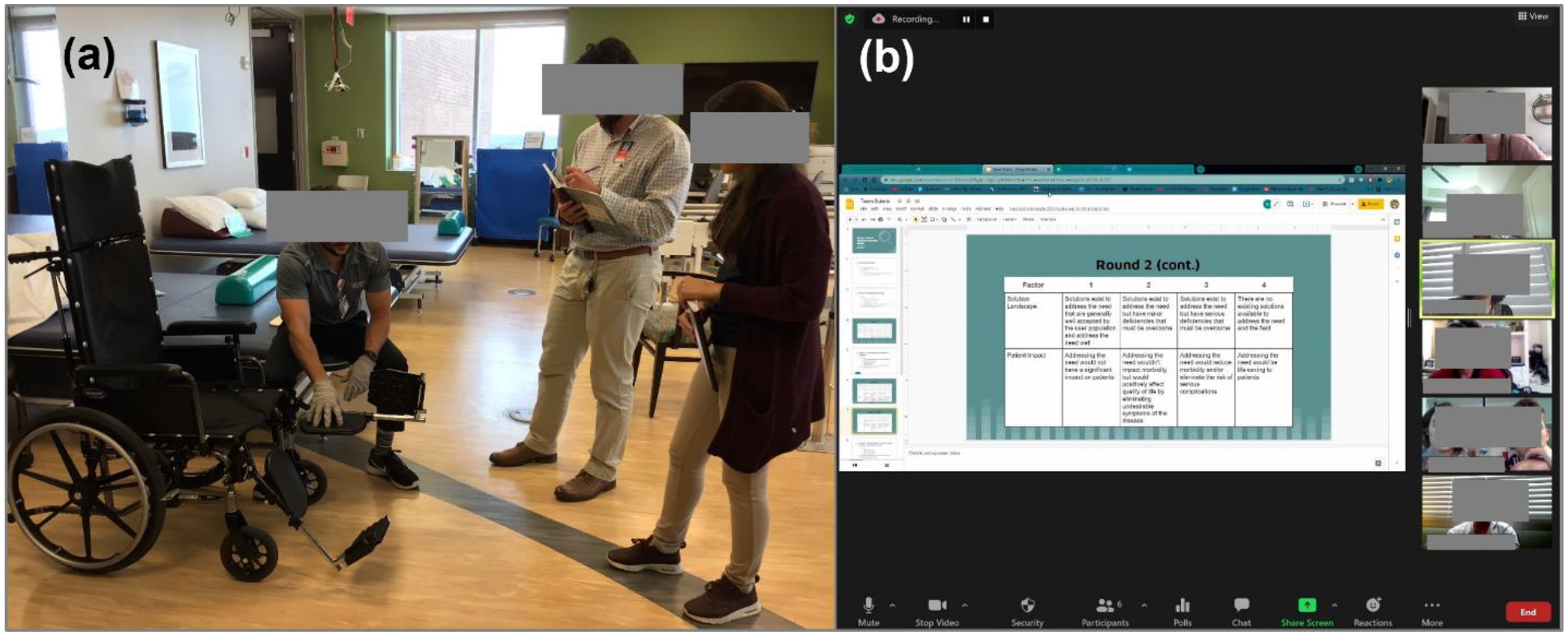
Examples of key CI activities (a) student mentor interaction at a rehabilitation center and (b) brainstorming sessions on clinical needs selection in course 2.

For course 2, there were three cohorts of six to eight students (total 20 students) in the three years. Due to the jump in enrollment in the course 1 in year 3, the number of students selected into course 2 was increased by 33% (from 6 to 8) while abiding by the project funding limitations. The clinical sites over the three years included adult and pediatric rehabilitation centers, an intensive hospital-based rehabilitation center, Department of Veterans Affair prosthetics clinic, a local prosthetics clinic, a sports medicine clinic, a general outpatient rehabilitation clinic, a rehabilitation clinic for underserved population and a pediatric communication disorders clinic. Figure 6 highlights the “observe and reflect” stages of experiential learning, a student team and clinical mentor interaction at a hospital-based rehabilitation center (Fig. 6a), and a student team virtual presentation and brainstorming session on clinical needs selection (Fig. 6b) with PT, medicine and engineering faculty.^5^

**Figure 7:**
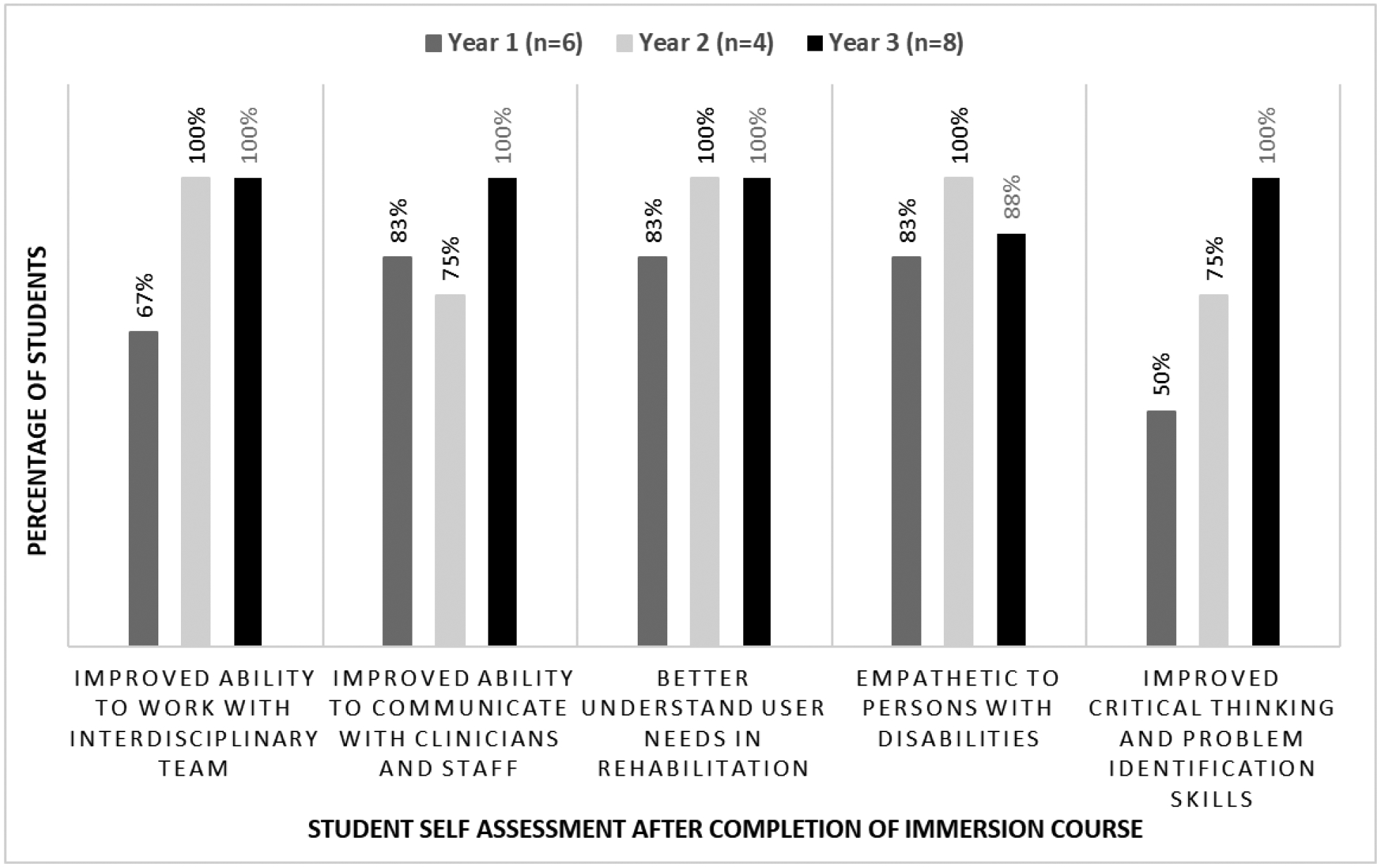
Exit survey data for course 2 on the course learning outcomes

Figure 7 shows exit survey response data for the second course with 90% of the students (three-year period) completing the survey. Students scored themselves strongly (50 to 100%) in the following course learning outcomes: improved ability to work with interdisciplinary professionals, improved ability to communicate with clinicians and staff, better understanding of user needs in rehabilitation practice, feeling of empathy toward people with disabilities and improved critical thinking and problem formulation skills. As demonstrated in Figure 7, a small improvement was observed in most criterion from year 1 to years 2 and 3, due to course refinements and adaptations.^6,14^ Inclusion of online resources and lecture videos on clinical immersion, training on methodical approaches in clinical needs identification and selection (adapted from the Stanford Biodesign process) and interprofessional interactions and discussions with clinical mentors and PT, medicine and engineering faculty during the immersion phase and during rotation presentations played a role in the positive outcomes.^13,14^

Each cohort of the course 2 students identified about forty clinical needs in RE that could have potential engineering solutions and that could benefit the disabled patients or the clinicians or the providers. These needs were further discussed for their design potential amongst the RE program faculty team and engineering design faculty and about three to four BME capstone design projects were pursued each year in the subsequent fall and spring semesters. Approximately 30 to 50% of the students in course 1 and course 2 participated in these RE design projects. Table I below lists six RE design projects offered through the program spanning different RE areas. RE design projects from year 3 cohort are in the discussion pipeline for future consideration as design projects.

In conclusion, the first three years of implementation of the BPRE program in a structured, experiential environment has been a success. Collaborative team teaching by medicine and PT faculty has played an integral role in this success. Building student interest in the underserved RE field through several course components and continued exposure to the field has been the backbone of this program. Indirect assessment methods such as student survey responses, enhanced student enrollment, student interest and participation in RE design projects has shown promising results in inspiring engineering students’ interest in RE and BME, influencing student career pathway decisions toward an engineering discipline with high clinical needs, in preparing engineering students to work in an interprofessional and collaborative clinical environment and in instilling engineering design thinking skills for creation of new RE technology based on real-world immersion. Challenges faced during the three years of implementation included enrolling students and mentoring them in completing the entire program, offering a standalone BME capstone course in the absence of a BME undergraduate program and COVID related delays in program implementation. Future program modifications will focus on addressing these challenges and will track student interests in the RE field until program completion along with further expansion and improvement of the program.

## Supplementary Material

Supplemental Table 2

Supplemental Table 1

## Figures and Tables

**Table I:** BME capstone projects completed through the experiential RE program

BME Capstone Project Description	Year
A passive flexion-extension board design for knee surgery patients	Year 1
An automated self-adjustable prosthetic socket design for transtibial amputees	Year 1
A low-cost accessible wheelchair transfer system design for paraplegic patients	Year 1
A partial hand and finger prosthetic design for transcarpal and metacarpal amputees	Year 1
A novel phone application design for speech therapy patients that also incorporates lip recognition	Year 2
A smart prosthetic design with cooling elements for transtibial amputees	Year 2
