# Supplementary material for: An Experiential Learning Based Design Program in Rehabilitation Engineering
Source: Biomed Eng Educ. Author manuscript; Available in PMC 2023 Oct 23. (PMC10593420; doi:10.1007/s43683-022-00091-2)
Supplement: Supplemental Table 2 [file NIHMS1864064-supplement-Supplemental_Table_2.docx]

Supplemental Table II: Post-course survey results for introductory RE course

| Student Self-Assessment Criteria | Strongly Agree - Agree  (% ) | | | Neutral  (% ) | | | Disagree - Strongly Disagree  (%) | | |
| --- | --- | --- | --- | --- | --- | --- | --- | --- | --- |
|  | *Year 1*  *(n =23)* | *Year 2*  *(n =40)* | *Year 3*  *(n =77)* | *Year 1*  *(n =23)* | *Year 2*  *(n =40)* | *Year 3*  *(n =77)* | *Year 1*  *(n =23)* | *Year 2*  *(n =40)* | *Year 3*  *(n =77)* |
| Consider RE as a career option after course completion | 95 | 76 | 77 | 4 | 20 | 16 | 1 | 7 | 7 |
| Increased interest in BME RE design projects after course completion | 65 | 61 | 58 | 30 | 30 | 30 | 5 | 9 | 12 |
| Increased Interest in a BME capstone course after course completion | 69 | 56 | 50 | 26 | 30 | 28 | 5 | 14 | 32 |
| Suitability of course content for RE careers | 87 | 98 | 96 | 13 | 2 | 3 | 0 | 0 | 1 |
