# Supplementary material for: An Experiential Learning Based Design Program in Rehabilitation Engineering
Source: Biomed Eng Educ. Author manuscript; Available in PMC 2023 Oct 23. (PMC10593420; doi:10.1007/s43683-022-00091-2)
Supplement: Supplemental Table 1 [file NIHMS1864064-supplement-Supplemental_Table_1.docx]

Supplemental Table I: Key elements of the introductory course in RE (course1)

| Modules | Learning Outcomes | Assessments |
| --- | --- | --- |
| Module-1: Introduction and Mobility Devices | - Learn key terminologies and current standards in RE and assistive technology.  - Understand the biomechanical principles of seating systems and engineering design basics of wheelchairs. | -Homework  -Discussion Post on Clinical Lectures |
| Module-2:  Prosthetic and Orthotic Devices | - Gain knowledge on the different upper and lower extremity prosthetic and orthotic components  - Understand the movement mechanics of amputees and the biomechanics of orthotic device design. | - Homework  - Discussion Post on Clinical Lectures  - Mid-term Exam |
| Module-3: Rehabilitation Robotics | - Understand the kinematics, design, safety and performance evaluation aspects of rehabilitation robots and exoskeletons. | -Homework  -Discussion Post on Clinical Lectures |
| Module-4:  Sensory Devices & Clinical Assessment | - Learn about sensory augmentation devices and the different clinical assessment tools in rehabilitation. | - Clinical Assessment  - Final Exam  - Term Project & Video |
